# Tumor necrosis factor inhibitors are associated with reduced complement activation in spondylarthropathies: An observational study

**DOI:** 10.1371/journal.pone.0220079

**Published:** 2019-07-23

**Authors:** Ingrid Hokstad, Gia Deyab, Morten Wang Fagerland, Torstein Lyberg, Gunnbjørg Hjeltnes, Øystein Førre, Stefan Agewall, Tom Eirik Mollnes, Ivana Hollan

**Affiliations:** 1 Lillehammer Hospital for Rheumatic Diseases, Lillehammer, Norway; 2 Institute of Clinical Sciences, University of Oslo, Oslo, Norway; 3 Department of Medical Biochemistry, Innlandet Hospital Trust, Lillehammer, Norway; 4 Oslo Centre for Biostatistics and Epidemiology, Research Support Services, Oslo University Hospital, Oslo, Norway; 5 Department of Medical Biochemistry, Oslo University Hospital Ullevål, Oslo, Norway; 6 Department of Medicine, Innlandet Hospital Trust, Lillehammer, Norway; 7 Department of Rheumatology, Oslo University Hospital, University of Oslo, Oslo, Norway; 8 Oslo University Hospital Ullevål, Oslo, Norway; 9 Department of Immunology, Oslo University Hospital, University of Oslo, Oslo, Norway; 10 Research Laboratory, Nordland Hospital, Bodø, Norway; 11 Faculty of Health Sciences, K.G. Jebsen TREC, University of Tromsø, Tromsø, Norway; 12 Centre of Molecular Inflammation Research, Norwegian University of Science and Technology, Trondheim, Norway; 13 Department of Medicine, Brigham and Women’s Hospital, Boston, Massachusetts, United States of America; Nippon Medical School Institute for Advanced Medical Sciences, JAPAN

## Abstract

**Background:**

The complement system is involved in pathogenesis of cardiovascular disease, and might play a role in accelerated atherogenesis in spondylarthropathies (SpA). Hence, we examined complement activation in SpA, and its relationship to antirheumatic treatment, inflammatory and cardiovascular markers.

**Methods:**

From PSARA, a prospective observational study, we examined 51 SpA patients (31 psoriatic arthritis (PsA), and 20 ankylosing spondylitis (AS)), starting tumor necrosis factor (TNF) inhibitor alone (n = 25), combined with methotrexate (MTX) (n = 10), or MTX monotherapy (n = 16). Complement activation was determined by the soluble terminal complement complex (sC5b-9), inflammation by erythrocyte sedimentation rate (ESR) and C-reactive protein (CRP), and endothelial function by finger plethysmography (Endopat) at baseline, after 6 weeks and 6 months of treatment.

**Results:**

SpA patients had sC5b-9 levels at (PsA) or above (AS) the upper limit of the estimated reference range. Median sC5b-9 levels decreased significantly from baseline to 6 weeks, with no significant difference between the AS and PsA group. Notably, a significant reduction in sC5b-9 was observed after administration of TNF inhibitor ± MTX, whereas no significant changes were observed in patients treated with MTX alone. Between 6 weeks and 6 months, sC5b-9 remained stable across all subgroups. Reduction in sC5b-9 was independently related to decreased ESR and CRP, and to increased high density cholesterol and total cholesterol. Reduction in sC5b-9 from baseline to 6 weeks was associated with improved EF in age and gender adjusted analyses.

**Conclusion:**

TNF-inhibition, but not MTX monotherapy, led to rapid and sustained reduction of complement activation in SpA. Thus, the observed decrease in cardiovascular morbidity in patients treated with TNF-inhibitors might be partly due to its beneficial effect on complement.

**Trial registration:**

Clinical Trials (NCT00902005), retrospectively registered on the 14^th^ of May 2009.

## Introduction

Spondylarthropathies (SpA), such as ankylosing spondylitis (AS) and psoriatic arthritis (PsA), are associated with increased cardiovascular (CV) morbidity and mortality [1–3], mainly due to premature atherosclerosis. The reason for this has not been fully elucidated yet, but immune dysregulation and inflammation appear to be important [4].

Indeed, inflammation is known to play a key role in atherothrombosis in general: from development of endothelial dysfunction (ED) and atheroma formation to atheroma destabilization and thrombosis [5]. Hence, the exaggerated inflammation in SpA might accelerate CV disease in these patients. To identify the exact pathways, and to clarify which immune factors mediate the pro-atherosclerotic processes, is essential for development of targeted CV therapy and prevention.

It is also important to improve insights into the pathophysiology of SpA, to limit disease activity and progression more effectively. Although genetic susceptibility, environmental triggers, and abnormal immune responses are known to be involved in the pathogenesis of SpA [6], the exact mechanisms are still unresolved. Intriguingly, the complement system, which is an essential component of the immune system, has been increasingly recognized as a substantial player in the development and progression of cardiovascular disease (CVD) [7].

The complement system’s main tasks are to protect the host against microbial invaders, to clear debris (such as apoptotic cells, and immune complexes), and to modulate inflammatory processes [8–10]. It is continuously undergoing a low-grade autoactivation, and can additionally be activated by antibodies and various pathogen-associated molecular patterns (PAMPs) and host damage-associated molecular patterns (DAMPs) expressed during tissue injury. PAMPs and DAMPs can induce infectious or sterile inflammation, respectively.

Regardless of pathway, complement activation triggers a cascade reaction, ultimately resulting in activation of C3 and C5, the latter with release of the potent anaphylatoxin C5a and formation of the terminal C5b-9 complement complex (TCC). TCC occurs in two forms, the soluble sC5b-9 and the membrane inserted membrane attack complex (MAC). sC5b-9 is virtually inert, but is an important and valuable marker of complement activation. MAC can cause lysis of Gram-negative bacteria and red cells. However, when nucleated cells are attacked by MAC, they frequently protect themselves, in a process called sublytic attack, which induce cellular activation. Further, this complex can activate the NLRP3 pathway, resulting in IL-1β and IL-18 production [11]. Though the complement system’s main task is to protect us from damaging pathogens, its powerful capability to trigger the immune system necessitates a strict control of its functions. Impaired regulation of this system may result in chronic inflammation, tissue damage, and increased susceptibility to specific autoimmune diseases, including vasculitis [12].

C5b-9 has been observed in human atherosclerotic arteries, with signs of increased complement activation in atherosclerotic plaque compared to healthy arterial tissue, and in ruptured compared to stable lesions [13]. Plasma sC5b-9 levels reportedly predict risk of death in patients with acute myocardial infarction [14], while C6 deficiency, making C5b-9 assembly impossible, has been found to reduce the size of atherosclerotic plaques by 50% in apoE−/− mice [15]. In rabbits, C5b-9 impairs endothelium-dependent vasorelaxation, and leads to ED, accelerated atherosclerosis, and premature death. C5b-9 has been reported to induce smooth muscle cell proliferation, and to increase monocyte chemoattractant protein-1 (MCP-1) secretion from human vascular smooth muscle cells. It also increases expression of adhesion molecules by endothelial cells, resulting in augmented leucocyte migration into the vessel wall through the activated endothelium. Furthermore, C5b-9 facilitates thrombogenesis by promoting platelet activation and aggregation, and increasing production of thrombin and fibrin [13].

Thus, one might expect that exaggerated complement activation in chronic inflammatory diseases, such as SpA, might augment CVD development. However, despite convincing evidence of the role of complement in inflammatory rheumatic diseases (IRDs) and atherosclerosis, and the widely recognized need to prevent premature CVD in IRDs [16], there is limited data available regarding complement activation in SpA. Moreover, to our knowledge, no studies have explored relationships between complement and CV risk in SpA so far.

In sum, data points to a vital role of complement in the development and progression of CVD. Therefore, in this study we wanted to examine the degree of complement activation in AS and PsA before and after initiation of antirheumatic treatment. We further examined associations between complement activation and markers of inflammation and CV risk, including endothelial function (EF).

## Patients and methods

### Patients

This observational prospective study is based on all SpA patients (31 PsA, and 20 AS) who completed 6 months follow-up in the PSARA (PSoriatic arthritis, Ankylosing spondylitis, Rheumatoid Arthritis) study, described in detail elsewhere [17]. Patients were recruited from October 2008 to May 2010. In brief, patients were included consecutively, as rheumatologists (not involved in the study) found indication for starting either Methotrexate (MTX) monotherapy (n = 16), or tumor necrosis factor inhibitor (anti-TNF) alone (n = 25) or combined with MTX (n = 10), due to active disease. Patients fulfilled either the Caspar criteria for PsA, or the modified New York criteria for AS. All patients were of Western European descent. Out of a total of 67 SpA patients included in the study, 16 patients discontinued the study due to adverse events (8), lack of effect (4), change in diagnosis (2), loss to follow-up (1), or Hepatitis C at inclusion (1).

### Data collection and assessment

Data collection included demographic data, medical history, life-style factors, medication, self-reported measures, physical findings and blood samples.

The patients were examined at baseline, after 6 weeks, and after 6 months of treatment.

### Complement activation

We assessed complement activity by measuring sC5b-9, using enzyme-linked immunosorbent assay (ELISA), as described in detail by Bergseth et al [18]. Values are presented as Complement Activation Units (CAU)/mL, with an estimated reference range of <0.7.

### Endothelial function

EF was determined by reactive hyperemia index (RHI) measured by finger plethysmography (Endopat). ED was defined as RHI <1.67 [19].

### Statistics

As most continuous variables were not normally distributed, results are presented as median and interquartile range (IQR). For comparison of continuous variables between two groups, independent samples t-tests (for normally distributed variables), or Wilcoxon signed rank test (for non-normally distributed variables) were applied. Distributions of categorical variables between groups were compared by Chi-square test or Fischer mid-p test, as appropriate. Wilcoxon one-sample signed rank test was applied to compare patient’s sC5b-9 levels to a reference value. Simple and standardized multiple linear regression analyses were applied to search for associations between baseline sC5b-9 and selected demographic, clinical and laboratory variables, including markers of disease activity, characteristics of SpA, cardiovascular risk (including RHI) and medication. We performed simple regression analyses to assess associations between baseline sC5b-9 and demographic data (age, gender), characteristics of rheumatic disease including inflammatory activity (C-reactive protein (CRP), white blood cells (WBC), erythrocyte sedimentation rate (ESR), hemoglobin (Hb), disease activity scores), and CV risk (established CVD, body mass index (BMI), hypertension, hyperlipidemia, diabetes, smoking status, alcohol consumption, exercise, RHI, and serum levels of total cholesterol, low-density lipoprotein (LDL-C), high-density lipoprotein (HDL-C)., HbA1c and homocysteine). As independent variables in multiple regression models, we included variables of specific clinical interest (age, gender, CRP, ESR, and RHI), as well as variables with a p-value <0.10 in simple regression analyses. Moreover, we evaluated if type of antirheumatic treatment (MTX monotherapy vs. anti-TNF regimens) or rheumatic diagnosis (PsA vs. AS), influenced sC5b-9 independently of inflammatory activity. Furthermore, we searched for relationships between change in sC5b-9 and change of the aforementioned parameters (if applicable) during treatment. Preliminary analyses were conducted to prevent violations of relevant assumptions. We performed several multiple adjusted analyses, to check if the presented data were consistent across multiple models, and therefore robust with respect to small changes of the independent variables. Results are based on complete-case analyses. P-values <0.05 were considered statistically significant. All tests were two-tailed. Statistical analyses were performed using SPSS 24.

### Ethics

The study was registered in “Clinical Trials” (NCT00902005), and the Norwegian Biobank register (2054). The Norwegian Regional Ethical Committee approved the study protocol (S-07377b), and all patients gave written informed consents.

## Results

### Patient characteristics

Baseline characteristics are shown in Table 1. Compared to PsA, AS patients were more likely to be smokers, and to use NSAIDs and statins. They also had a higher frequency of established CVD, a greater proportion of men (as expected, due to known male predominance in AS), and had lower LDL-C and HDL-C.

**Table 1:** Baseline characteristics.

	PsA (n = 31)	AS (n = 20)	P-value
Age, years	50 (41–59)	49 (41–57)	0.82
Gender, men; n (%)	18 (58)	16 (80)	0.11
Disease duration, years	2 (-4.4–8.4)	2.5 (-0.6–3.1)	0.001
sC5b-9 (CAU/mL)	0.7 (0.5–0.9)	0.8 (0.51–1.09)	0.43
**Antirheumatic treatment**:			
Anti-TNF; n (%)	5 (16)	20 (100)	<0.001
MTX; n (%)	16 (52)	0	<0.001
Anti-TNF + MTX; n (%)	10 (32)	0	<0.001
**CV risk factors:**			
ED; n (%)	9 (30)	9 (45)	0.28
RHI	2 (1.63–2.38)	1.7 (1.45–1.95)	0.06
Current smoker; n (%)	7 (23)	10 (50)	0.04
Hypertension; n (%)	7 (23)	6 (30)	0.79
BMI	26 (23–29)	28 (24–32)	0.34
Established CVD; n (%)	1 (3)	5 (25)	0.03
Cholesterol total (mmol/L)	5.3 (4.9–5.8)	5 (4.1–5.9)	0.03
LDL-C (mmol/L)	3.4 (2.9–4.0)	2.8 (2.2–3.4)	0.02
HDL-C (mmol/L)	1.3 (1.0–1.6	1.3 (1.2–1.5)	0.5
Triglycerides (mmol/L)	1 (0.5–1.5)	1.3 (1.0–1.7)	0.28
HbA1C	5.6 (5.4–5.9)	5.6 (5.3–6.0)	0.98
**Disease activity:**			
BASMI	NA	4.5 (4.25–4.75)	NA
BASDAI	4.3 (2.9–5.8)	5.3 (3.6–7.0)	0.11
BASFI	3.2 (1.7–4.8)	4.1 (2.4–5.8)	0.09
MHAQ	0.4 (0.2–0.6(	0.43 (0.21–0.66)	0.56
Physician global	2.1 (1.1–3.2)	2.6 (1.4–3.8)	0.38
Patient global (VAS)	4.3 (2–7.6)	5.6 (3.5–7.8)	0.62
WBC (10^09 /L)	6.4 (4.7–8.1)	7.9 (6.7–9.1)	0.08
ESR (mm/h)	7.5 (1.5–13.5)	9.5 (4.5–14.5	0.42
CRP (mg/L)	5 (0.8–9.3)	7.5 (1.6–13.4)	0.43
**Comedication:**			
Statin; n (%)	1 (3)	7 (35)	0.004
Acetylsalicylic acid; n (%)	2 (7)	2 (10)	0.37
NSAIDS; n (%)	15 (47)	14 (70)	0.13
ACE-inhibitor /AT2; n (%)	4 (14)	4 (20)	0.41
Systemic corticosteroid; n (%)	3 (10)	2 (10)	1.0
CCB; n (%)	2 (7)	2 (10)	1.0

All values are given as median (interquartile range), unless otherwise specified.

Established CVD is defined as previous presence of any of these conditions: Angina pectoris, stroke, myocardial infarction, carotid stenosis, chronic heart failure, claudicatio intermittens, aortic aneurysm, aortic regurgitation.

Abbreviations: PsA: psoriatic arthritis, AS: ankylosing spondylitis, MTX: Methotrexate, RHI: reactive hyperemia index, BMI: body mass index, BASMI: Bath Ankylosing Spondylitis Metrology Index, BASDAI: Bath Ankylosing Spondylitis Disease Activity Index, BASFI: Bath Ankylosing Spondylitis Functional Index, MHAQ: Modified Health Assessment Questionnaire, PhGA: Physician’s Global Assessment, PtGA: Patient’s Global Assessment, WBC: white blood cells, ESR: erythrocyte sedimentation rate, CRP: C-reactive protein, LDL-C: low-density lipoprotein cholesterol, HDL-C: high-density lipoprotein cholesterol, NSAIDs: non-steroidal anti-inflammatory drugs, ACE-inhibitor/AT2: angiotensin converting enzyme inhibitors/angiotensin II receptor antagonists, CCB: calcium channel blocker, NA: not applicable

### sC5b-9 levels at baseline

The total patient cohort had median baseline sC5b-9 levels at 0.7 CAU/mL, i.e. above the estimated reference value (p = 0.03), with no significant difference between PsA and AS patients. There was no baseline difference in sC5b-9 between smokers and non-smokers (p = 0.44).

### Effects of antirheumatic treatment

**Fig 1:**
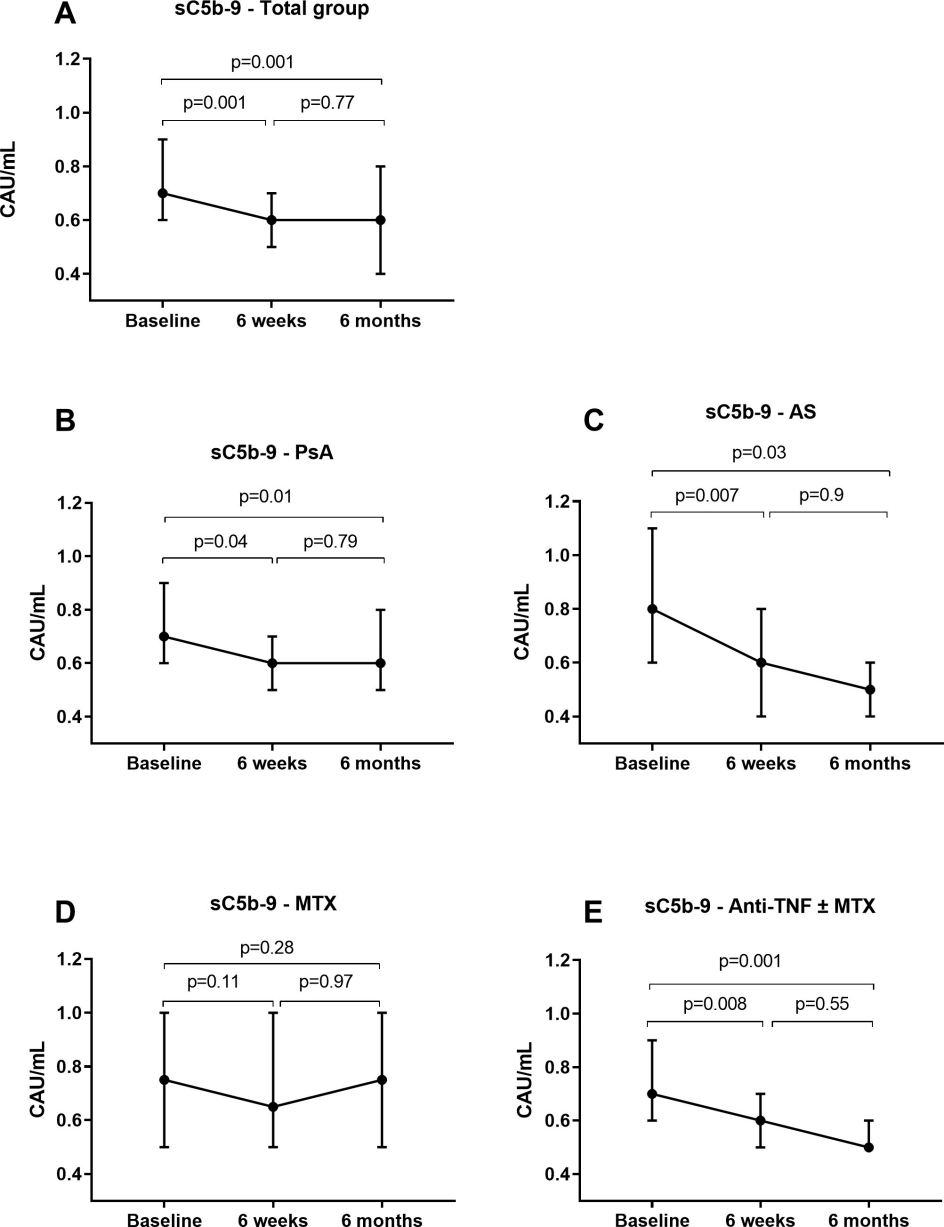
Change in complement activation with treatment. sC5b-9 levels in CAU/ml at baseline, after 6 weeks and after 6 months of treatment for the whole study group (A), and for PsA (B), AS (C), MTX (D) and TNF ± MTX (E) subgroups. Points represent median value, while error bars show 95% confidence interval. Abbreviations: PsA: psoriatic arthritis, AS: ankylosing spondylitis, MTX: methotrexate, TNF: tumor necrosis factor inhibitor, CAU: complement activation units.

In both disease groups, sC5b-9 decreased significantly during the first 6 weeks of treatment (mean difference _base– 6 weeks_ 0.14 CAU (95% CI 0.07–0.22, p = 0.001, Fig 1).

Between 6 weeks and 6 months, sC5b-9 levels remained relatively stable in the PsA group, while in the AS group sC5b-9 continued to decrease (but the difference between 6 weeks and 6 months visit was not statistically significant in any of the two groups).

Both anti-TNF ± MTX, and MTX monotherapy was associated with reduction in sC5b-9 after 6 weeks, though this effect was statistically significant in the anti-TNF ± MTX group only. Between 6 weeks and 6 months, sC5b-9 level continued to decrease in the anti-TNF ± MTX group, but the difference was not statistically significant. We assessed change in sC5b-9 levels in PsA patients using anti-TNF ± MTX separately, to see if the effect of anti-TNF was consistent across diagnostic subgroups, and found that anti-TNF ± MTX was still associated with improved sC5b-9 (p _baseline to 6 months_ = 0.005. P _baseline—6 weeks_ = 0.18, p _6 weeks—6 months_ = 0.87, respectively). In the MTX group sC5b-9 levels increased non-significantly between 6 weeks and 6 months, and at the 6 months visit they equaled baseline levels.

Compared to the whole population, the decrease in sC5b-9 levels between baseline and 6 weeks was almost three times greater in patients with sC5b-9 above the upper quartile at baseline (7 AS and 6 PsA patients; mean difference _base– 6 weeks_ 0.37 CAU (95% CI 0.17–0.57, p = 0.007; p _6 weeks– 6 months_ = 0.53, and p _base– 6 months_ = 0.054).

The size of the reduction was similar in patients receiving anti-TNF ± MTX (n = 10) and those receiving MTX monotherapy (n = 3).

There were no significant differences in changes of sC5b-9 levels between smokers and non-smokers at any point of time (neither for the whole patient cohort, nor when assessing treatment groups or diagnostic groups separately).

We found no interaction effects between the antirheumatic treatment regimens and diagnoses.

CRP and ESR improved from baseline to 6-week and 6-month visits, as shown in Table 2. However, there was no significant improvement in RHI at any of the time points either in the total population, or in any of the subgroups.

**Table 2:** Changes in selected variables during treatment.

	Psoriatic Arthritis (n = 31)			Ankylosing Spondylitis (n = 20)			TNF-inhibitor (n = 35)			MTX monotherapy (n = 16)		
	Baseline	6 w	6 m	Baseline	6 w	6 m	Baseline	6 w	6m	Baseline	6 w	6 m
sC5b-9 (CAU/mL)	0.7	0.6*	0.6	0.8	0.6**	0.5	0.7	0.6**	0.5	0.8	0.6	0.7
CRP (mg/L)	5.0	3.0*	2.0	7.5	1.0*	1.0	5.0	1.0**	1.0	7.5	5.5*	2.0*
ESR (mm/h)	7.5	6.5	6.0	9.5	3.5***	3.0	8.5	3.0***	3.5	8.0	9.5	6.0
WBC (10^09 /L)	6.4	5.9*	5.5	7.9	6.7**	6.4	7.3	6.5**	6.3	6.4	5.9	4.9*
RHI	2.0	1.9	1.8	1.7	1.8	1.9	1.8	1.9	1.8	2.0	1.9	1.9
Cholesterol(mmol/L)	5.3	5.4	5.6	5.0	5.3**	5.2	5.1	5.3	5.2	5.3	5.6	6.2
LDL (mmol/L)	3.4	3.5	3.6	2.8	3.1*	3.1	3.1	3.3	3.0	3.5	3.5	3.8
HDL (mmol/L)	1.3	1.4	1.3*	1.3	1.4**	1.4	1.3	1.5**	1.4	1.4	1.4	1.3**
TRG (mmol/L)	1.0	1.1	1.2	1.3	1.3	1.2	1.2	1.2	1.2	0.9	1.2*	1.2
HbA1C	5.6	5.5*	5.3	5.6	5.6	5.7	5.6	5.5	5.6	5.6	5.5	5.4
BASDAI	4.3	1.9**	2.1	5.3	2.2***	2.5	5.0	2.0***	2.5	4.5	2.1*	2.1
MHAQ	0.4	0.3***	0.3	0.4	0.3*	0.3	0.4	0.3***	0.3	0.4	0.3**	0.3
Phys Global	2.1	1.4**	0.8*	2.6	1.5***	0.9	2.1	1.4***	0.9**	2.7	2.1**	1.2
Pat Global	4.3	1.9***	1.4	5.6	1.5**	1.4	4.6	1.5*	1.4	4.9	1.9*	2.3*

Values are given as median. Significant changes with a p-value < 0.05 is marked with *, p = 0.001–0.01 = **, p<0.001 = ***.

Abbreviations: PsA: psoriatic arthritis, AS: ankylosing spondylitis, TNF: Tumor Necrosis Factor inhibitor (with or without MTX co-medication), MTX: Methotrexate, CRP: C-reactive protein, ESR: erythrocyte sedimentation rate, WBC: white blood cells, RHI: reactive hyperemia index, LDL: low-density lipoprotein cholesterol, HDL: high-density lipoprotein cholesterol, TRG: Triglycerides, BASDAI: Bath Ankylosing Spondylitis Disease Activity Index, MHAQ: Modified Health Assessment Questionnaire, Phys global: Physician’s Global Assessment, Pat Global: Patient’s Global Assessment

### Relationships between sC5b-9 and selected clinical and laboratory variables

In simple regression analyses, only ESR and Hb were related to sC5b-9 at baseline (Table 3). In age and gender adjusted analyses, there was a significant relationship between baseline Hb and sC5b-9 levels. However, after adjusting for CRP, WBC and ESR, this association was no longer significant. In adjusted models, both ESR and CRP were significantly related to sC5b-9 levels at baseline (Table 3). There were no significant associations between baseline sC5b-9 and baseline RHI in adjusted analyses (Table 3).

**Table 3:** Relationship between baseline sC5b-9 and selected clinical and laboratory variables.

	Unadjusted analyses			Adjusted analyses Model 1			Adjusted analyses Model 2		
	B	95% CI	p	B	95% CI	p	B	95% CI	p
Age	0.005	-0.003, 0.012	0.23	0.004	-0.004, 0.012	0.27	0.002	-0.006–0.009	0.65
Gender	0.044	-0.157, 0.245	0.66	−0.033	-0.173, 0.239	0.75	-0.10	-0.302–0.102	0.32
RHI	−0.148	-0.315, 0.019	0.08	−0.027	-0.3250, 0.195	0.81			
ESR	0.010	0.004, 0.016	0.001				0.018	0.004, 0.032	0.02
WBC	0.042	-0.003, 0.088	0.07				0.022	-0.029, 0.072	0.39
CRP	0.003	-0.001, 0.007	0.11				−0.008	-0.014, -0.001	0.03
Hb	−0.089	-0.151, -0.027	0.006				−0.041	-0.131, 0.049	0.36

Abbreviations: B: beta, p: p-value, RHI: reactive hyperemia index, ESR: erythrocyte sedimentation rate, WBC: white blood cells, CRP: C-reactive protein, Hb: Hemoglobin.

In unadjusted analysis, changes in sC5b-9 from baseline to 6 weeks were positively related to changes in markers of disease activity (ESR, WBC and CRP), and negatively to changes in HDL-C and total cholesterol levels (Table 4).

**Table 4:** Relationships between changes in sC5b-9 during the first 6 weeks of treatment and selected clinical and laboratory parameters.

	Unadjusted analyses			Adjusted analyses											
				Model 1			Model 2			Model 3			Model 4		
	B	95% CI	p	B	95% CI	p	B	95% CI	p	B	95% CI	p	B	95% CI	p
Age	0.005	-0.003, 0.012	0.23	0.004	-0.002, 0.01	0.16	0.002	-0.004, 0.008	0.89	0.003	-0.003, 0.010	0.32	0.003	-0.003, 0.009	0.31
Gender	0.044	-0.157, 0.245	0.66	−0.031	-0.197, 0.135	0.71	-0.10	-0.25, 0.051	0.24	-0.060	-0.221, -0.101	0.45	-0.077	-0.222, 0.069	0.29
RHI Δ	−0.143	-0.315, 0.019	0.08	−0.166	-0.33, 0.001	0.05							-0.096	-0.244, 0.054	0.21
ESR Δ	0.01	0.006, 0.017	0.001				0.01	0.001, 0.019	0.03				0.01	0.004, 0.016	0.001
CRP Δ	0.005	-0.002, 0.008	0.002				0.001	-0.003, 0.006	0.59						
HDL Δ	−0.320	-0.566, -0.075	0.01							-0.310	-0.560, -0.061	0.02	-0.084	-0.335, 0.168	0.51
Chol Δ	-0.165	-0.291, -0.038	0.01												

Δ indicates change from baseline to 6 weeks. In model 2, CRP was significantly related to sC5b-9 after adjustments for age and gender(p = 0.001), but this association disappeared when adjusting for ESR. When adjusting all models for diagnosis and treatment, the association with HDL was significant in model 3 (p = 0.045), while in model 1 RHI was not significant (p = 0.063). When assessing the relationship between sC5b-9 reduction and ESR, this association remained significant when adjusting for age, gender, diagnosis and treatment; p for ESR = 0.001. Total cholesterol was not associated to sC5b-9 in any adjusted models (Data can be found in supplementary file “S1 File”).

Abbreviations: B: beta, p: p-value, RHI: reactive hyperemia index, ESR: erythrocyte sedimentation rate, CRP: C-reactive protein, HDL-C: high-density lipoprotein cholesterol, Chol: total cholesterol.

At six weeks, changes in sC5b-9 were related to changes in RHI, independently of age and gender. However, after adjustments for changes in ESR and HDL-C, only ESR remained significantly associated to changes in sC5b-9 (Table 4). Changes in Hb during treatment were not related to changes in sC5b-9 at any point of time.

## Discussion

Our study revealed that patients with active SpA had complement activation levels at (PsA) or above (AS) the upper limit of the estimated reference range. Importantly, complement activation decreased with anti-TNF treatment already within 6 weeks. Patients with the highest sC5b-9 baseline levels had a reduction in sC5b-9 from baseline to 6 weeks that was almost three times larger than the mean reduction of sC5b-9 in the whole group. This suggests that the relatively modest sC5b-9 reduction we observed in the whole group reflects the fact that half of our patients had complement activation levels within the normal reference range at baseline. In the total cohort, the beneficial effect of antirheumatic treatment was significantly apparent also after 6 months. However, subanalyses showed that after 6 weeks, complement activation continued to decrease only in the anti-TNF± MTX group, while in MTX treated patients, complement activation returned to baseline levels. This corresponded with a continued reduction in sC5b-9 from 6 weeks to 6 months in AS patients, whereas in PsA patients sC5b-9 remained stable during the same period; possibly reflecting that all AS patients were treated with anti-TNF regimens, while more than half of the PsA patients received MTX monotherapy. Correspondingly, there were no significant differences in the magnitude of change in complement activation between PsA patients using anti-TNF and AS patients at any point of time. sC5b-9 levels remained above the estimated reference range during the whole study for patients treated with MTX monotherapy.

Similar to our findings, previous studies have reported elevated plasma sC5b-9 levels in IRDs, such as PsA and rheumatoid arthritis (RA) [20,21]. While several studies have shown complement activation in synovial fluid of RA patients [22,23], knowledge about local complement activation in organs and tissues affected by inflammation in SpA is limited. Whether the complement complexes we found were produced systemically, or represented a spillover from local production, remains unknown. Notably, there might be discrepancies between plasma sC5b-9 levels and sC5b-9 levels locally in affected joints, vessels, or other extra-articular structures. Furthermore, the membrane attack complex is inserted in cells and membranes locally in the organ and give damage to the tissue, whereas sC5b-9 in synovial fluid and plasma is only a biomarker indicating that complement activation with its potential harm is going on.

We do not know the cause of complement activation in our patients. To identify which complement pathways are activated in SpA might provide some clues as to whether disease activity in SpA is antibody-driven, or triggered by other factors, such as damaged self-structures that are exposed in the diseases tissue, or by microbial agents; both which may activate the classical and the lectin pathway in the absence of antibodies. However, our laboratory methods were not suited to identify which complement pathway was activated in our patients. Studies investigating activation of different complement pathways in SpA are scarce, but one article revealed increased C1/C1-inhibitor complex levels in PsA, suggesting antibody-initiated activation of the classical pathway [20]. Notably, there are still controversies as to whether PsA and AS are mainly autoimmune (i.e. antibody-driven) or autoinflammatory diseases (i.e. originating from an abnormal and excessive immune response) [6].

As there is limited clinical experience regarding the reference range of sC5b-9, and the half-life of sC5b-9 is short (approximately one hour), the interpretation of sC5b-9 levels can be challenging. More precisely, we do not know for sure how complement activation fluctuates short-term in our patients, or how large changes in sC5b-9 levels that would represent a clinically meaningful improvement [18].

An abundance of clinical trials has reported anti-inflammatory effects of anti-TNF and/or MTX, and one would therefore expect that markers of complement activation, similar to other inflammatory biomarkers such as ESR or CRP, would be reduced by these drugs. While our results support this notion, the association we observed between antirheumatic treatment and reduced complement activation, was not independent of reduction in CRP and ESR. Still, to our knowledge, this study is the first to demonstrate an association between anti-TNF and sC5b-9 in patients with AS and PsA. In support of our findings, anti-TNF drugs have been previously reported to inhibit the classical complement pathway in inflammatory bowel disease [24], and to decrease serum levels of C3 and C4 in PsA and RA [25,26]. Moreover, TNF levels have been found to be correlated with sC5b-9 levels in RA, indicating a plausible association between TNF-inhibition and reduced complement activity [23].

Details of how anti-TNF and MTX interact with the complement system are not fully understood. One theoretical explanation could be that anti-TNF reduces activation of the classical pathway due to inhibition of IL-6 [27], which consequently reduces CRP synthesis, hindering it from triggering complement activation [28]. This notion is in line with the observed association between complement activation and CRP in our study. Also, given that TNF increases hepatogenic production of complement factors [29], anti-TNF administration could theoretically reduce complement synthesis in the liver. Furthermore, antirheumatic treatment would be expected to dampen systemic inflammation, thereby lowering levels of inflammatory mediators overall. It is tempting to speculate that anti-TNF treatment reduces inflammation locally in joints and other organs, and that this reduces the DAMP load, which subsequently will reduce activation of both the classical, and in particular, the lectin pathway. Regarding MTX’s direct effect on complement activation, little evidence is available. Nevertheless, MTX is known to reduce production of TNF and other pro-inflammatory components of the immune system [30], and its overall anti-inflammatory effect might also attenuate the complement cascade. Interestingly, the recent results from the Cardiovascular Inflammation Reduction Trial (CIRT) found that MTX did not reduce either CRP, Interleukin-6, Interleukin-1 β, or CV events compared to placebo [31]. Though this study did not involve patients with rheumatic disease or a high grade of inflammation, it challenges the current understanding of MTX’s anti-inflammatory properties.

In our study, reduction in sC5b-9 was related to improvement in EF after 6 weeks of treatment, independently of age and gender. This improvement was not independent of changes in ESR and CRP. From 6 weeks to 6 months, sC5b-9 levels overall did not change further, and we saw no relationship with EF in this period. At baseline, there was no significant association between sC5b-9 and EF and other factors related to increased CV risk, including traditional CV risk factors. Nevertheless, previous research provides evidence for the role of complement in CVD. For example, sC5b-9 levels have been found to be related to circulating markers of ED [32], such as E-selectin, vascular adhesion molecule-1, and von Willebrand factor. Furthermore, there is convincing evidence that complement activation promotes prothrombotic and proinflammatory properties of endothelial cells [13]. The lack of association between sC5b-9 and EF at baseline and after 6 months in our study might simply reflect an actual non-existent relationship, or it could be due to Type-II error or our method for evaluation of EF. Finger plethysmography mainly assesses endothelium-dependent vasodilation in microvasculature, reflecting different pathophysiological processes in the vascular tree than other approaches for EF assessment [33]. An advantage of our method is its non-invasive character. Moreover, several large studies have demonstrated that ED, measured by finger plethysmography, can predict CV events, and correlates with CV risk factors, such as coronary ED [34,35]. An alternative approach could have been flow-mediated dilation (FMD) of the brachial artery, although this method is technically more challenging, and requires extensive operator training [36].

The AS group had double the proportion of smokers compared to the PsA group, but we found no differences in baseline sC5b-9 levels or changes in sC5b-9 with treatment between smokers and non-smokers. In line with this, one study found no change in sC5b-9 expression in sera exposed to tobacco smoke extract, despite signs of upstream complement activation [37]. In contrast, a few other in vitro studies have found indications of alternative complement pathway activation following tobacco exposure [38,39]. It is unknown whether the results from these in vitro studies translates to the effect smoking might have on complement activation in vivo. Also, it is worth noting that our patients were instructed not to smoke in the 12 hours prior to examination. Thus, we were not likely to see any acute effects of smoking on sC5b-9 levels.

Summarized, there are strong scientific indications that complement activation is heavily involved in CVD, and the association—although weak—we found between reduced complement activity and improved EF, could support this theory. These results should be interpreted with caution, and needs to be validated in future research.

Changes in sC5b-9 levels after treatment were inversely related to changes in HDL-C and total cholesterol levels. This is in line with previous studies demonstrating an inverse relationship between sC5b-9 and HDL-C [40]. This might be explained by the relationship between complement activation and inflammation, as both HDL-C and total cholesterol are known to increase with decreasing disease activity in IRDs [41]. Indeed, several studies have shown that SpA patients treated with TNF-inhibitors, increase their HDL-C levels [41,42]. It has long been known that HDL-C can prevent the lytic actions of sC5b-9 [43], and more recently, it has been demonstrated that HDL-C carries proteins that inhibit complement activation [44].

There are several limitations to our study. First, the small sample size increases the risk of Type-Ⅱ errors. Secondly, the observational design obviously does not allow for any certain conclusions regarding causal relationships between antirheumatic treatment and reduced complement activation. However, to minimize effects of potential confounders, we adjusted for them by statistical methods. Despite these potential shortcomings of observational studies, the study design was chosen of ethical reasons, to ensure adherence to national treatment guidelines, and to make certain that all patients received optimal, recommended medication. Observational studies also allow immediate treatment to all study participants, they reflect a real-world clinical setting, and are more feasible than randomized controlled trials (RCTs), whilst often generating similar effect estimates as RCTs [45]. Nevertheless, the lack of randomization regarding treatment, combined with the disparities in pathophysiology in AS and PsA, makes direct comparison of treatment effect challenging.

An advantage of our work is the novelty of measuring complement activation in AS patients, its comparison to PsA, and assessing the effect of antirheumatic treatment on complement activation in a well-characterized patient population. Also, as there is limited experience in measuring and evaluating complement activation in general, our study adds new valuable insights to this area.

## Conclusions

SpA patients had sC5b-9 levels at or above the upper limit of the estimated reference range. Complement activation decreased significantly with anti-TNF treatment within 6 weeks. The effect of antirheumatic treatment on complement activation seemed to be better in AS than in PsA, possibly reflecting differences in treatment regimens for the two diseases. Reduction in sC5b-9 from baseline to 6 weeks was associated with improved EF in age and gender adjusted analyses. In theory, the observed decrease in CVD morbidity in patients treated with antirheumatics might be partly due to its beneficial effect on complement activation. Further research is needed to clarify if markers of complement activation might represent useful biomarkers of cardiovascular risk and therapeutic response in SpA, and to investigate the potential link between complement activation and EF. Future implications of our research might be development of novel treatment methods in SpA, where inhibition of the complement system might provide an alternative that potentially could reduce disease activity and CV risk in these patients. The challenge lies in preserving the complement systems’ crucial protective properties, while at the same time preventing its potentially harmful effects.

## Supporting information

S1 File **S1 File.sav**: SPSS file containing all data.
(SAV)
